# Supplementary material for: The Hydrophobin-Like OmSSP1 May Be an Effector in the Ericoid Mycorrhizal Symbiosis
Source: Front Plant Sci. 2018 May 1;9:546. doi: 10.3389/fpls.2018.00546 (PMC5938622; doi:10.3389/fpls.2018.00546)
Supplement: Supplementary file 1 [file Data_Sheet_1.docx]

Supplementary Material

The hydrophobin-like OmSSP1 may be an effector in the ericoid mycorrhizal symbiosis

Salvatore Casarrubia, Stefania Daghino, Annegret Kohler, Emmanuelle Morin, Hassine-Radhouane Khouja, Yohann Daguerre, Claire Veneault-Fourrey, Francis M. Martin, Silvia Perotto, Elena Martino*

*** Correspondence:** Elena Martino: [elena.martino@unito.it](mailto:elena.martino@unito.it)

# Supplementary Figures and Tables

##
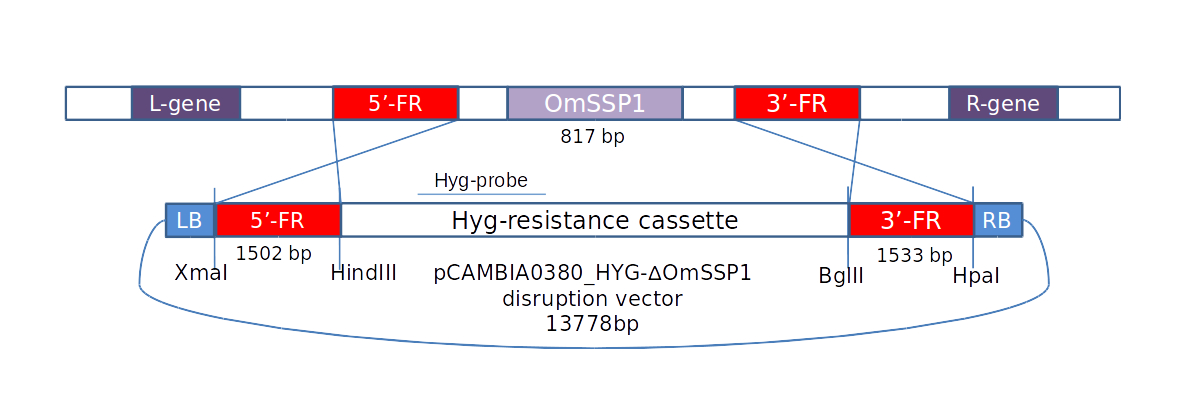
Supplementary Figures

**Supplementary Figure 1.** pCAMBIA0380_HYG-ΔOmSSP1 scheme representing the region involved in the homologous recombination. 5’-FR and 3’-FR: 5’and 3’ flanking regions; LB and RB: left and right borders of the T-DNA; Hygromycin-resistance cassette containing the *A. nidulans* gpdA promoter, the *E. coli* hph gene, and the *A. nidulans* trpC gene terminator region derived from the pAN7-1 plasmid (Punt et al., 1987); Hyg-probe: region probed for the Southern blot analysis; L- and R-gene: genes flanking the OmSSP1 in the genome.


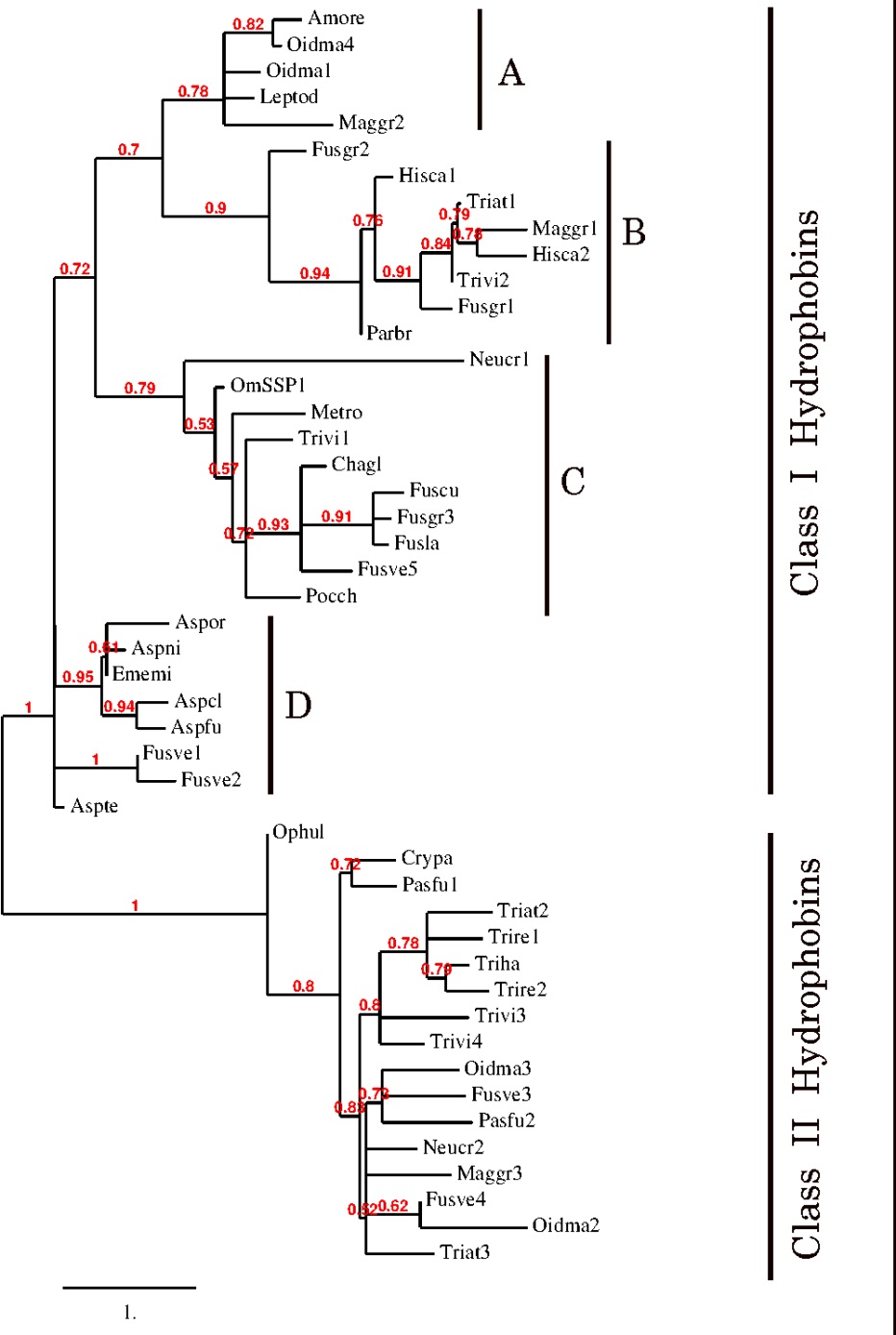


**Supplementary Figure 2. Phylogenetic tree of OmSSP1 and *O. maius* hydrophobins with other annotated hydrophobins from Ascomycetes.** The analysis included protein sequences annotated or described as Ascomycetes class I and class II hydrophobins (listed in Table S3), OmSSP1 and the four *O. maius* hydrophobins. This sequence alignment did not considered the amino acid sequence comprise between C3 and C4. Muscle algorithm implemented in MEGA7 (Tamura et al., 2007) was used to generate the multiple protein sequence alignment. The phylogenetic tree was reconstructed on the Phylogeny.fr platform (Dereeper et al., 2008) using the maximum likelihood method (Guindon & Gascuel 2003) implemented in the PhyML program (v3.1/3.0 aLRT). The WAG substitution model was selected assuming an estimated proportion of invariant sites (of 0.153) and 4 gamma-distributed rate categories to account for rate heterogeneity across sites. The gamma shape parameter was estimated directly from the data (gamma=6.274). Reliability for internal branch was assessed using the aLRT test (SH-Like). Graphical representation and edition of the phylogenetic tree were performed with TreeDyn (v198.3; Chevenet et al., 2006).


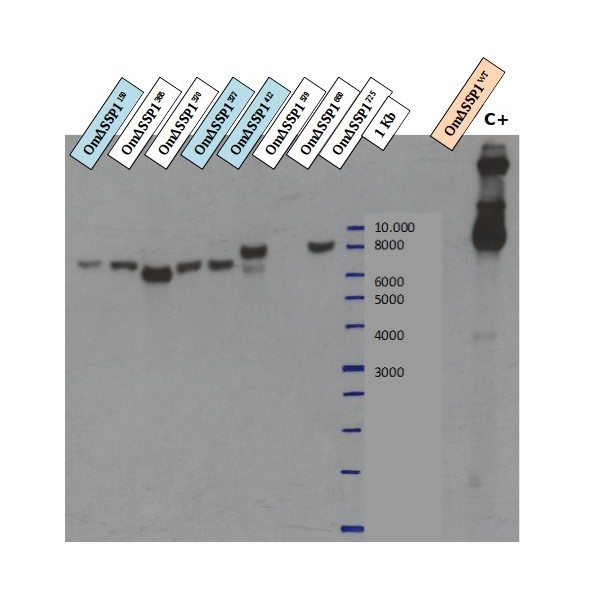


**Supplementary Figure 3. Southern blot analysis indicated that 4 mutants have a single insertion.** Eight putative homologous recombinants OmΔSSP1^150^, OmΔSSP1^368^, OmΔSSP1^370^, OmΔSSP1^377^, OmΔSSP1^412^, OmΔSSP1^579^, OmΔSSP1^660^ and OmΔSSP1^725^ out of the 742 screened transformants were identified. Their genomic DNA together with the WT strain DNA were digested with the BamHI enzyme for the Southern hybridization analysis. Four out of the eight candidates (OmΔSSP1^150^, OmΔSSP1^377^, OmΔSSP1^368^, OmΔSSP1^412^) showed a single band at the expected size. WT strain genomic DNA was used as negative control, whereas the plasmid PCAMBIA-0380-Hyg containing the deletion cassette was used as positive control.


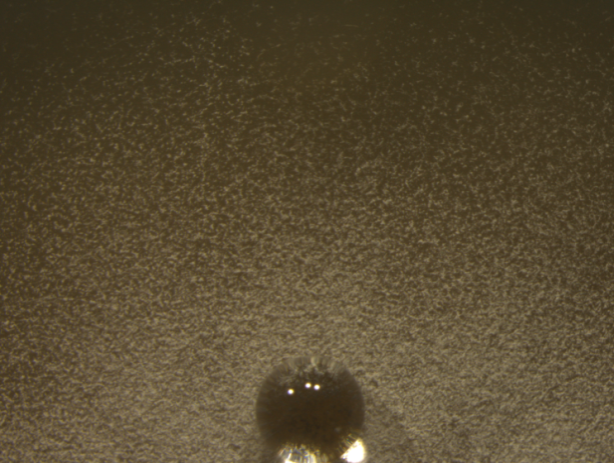

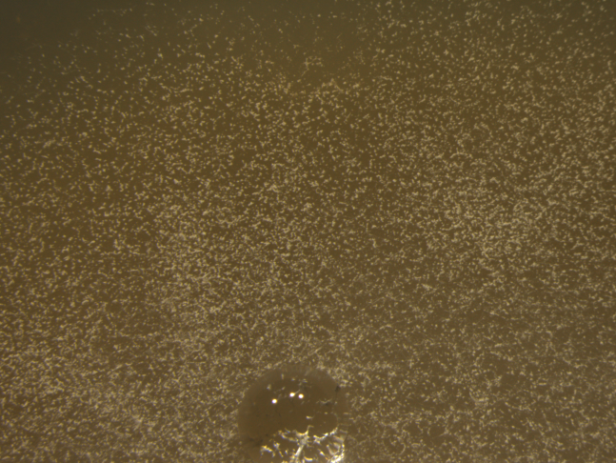

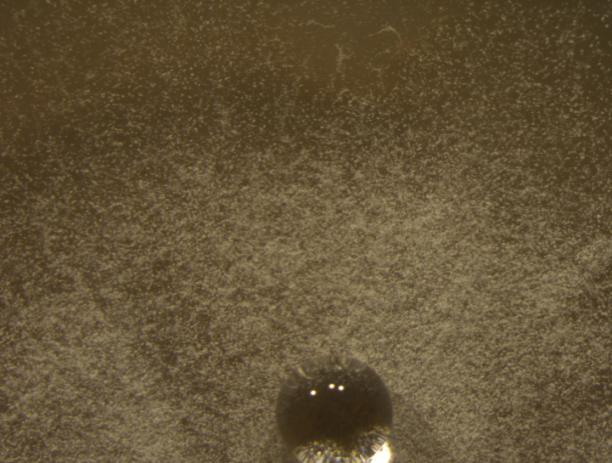

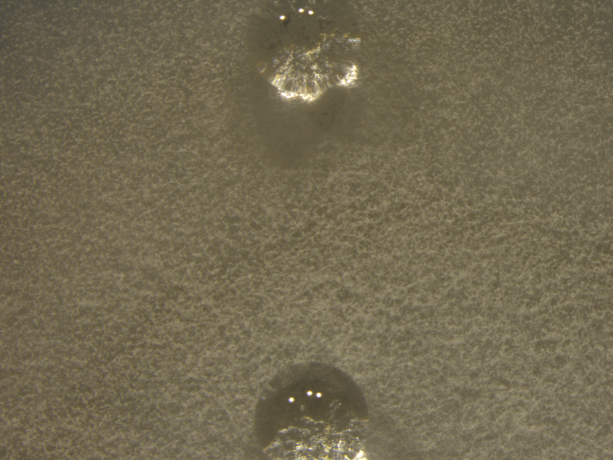


**(A)**

**(D)**

**(C)**

**(B)**

Supplementary Figure 4. OmΔSSP1 mutants do not show a modified wettability phenotype as compared to the *O. maius* WT: (A) *O. maius* WT, (B) OmΔSSP1^150^, (C) OmΔSSP1^377^, (D) OmΔSSP1^412^.


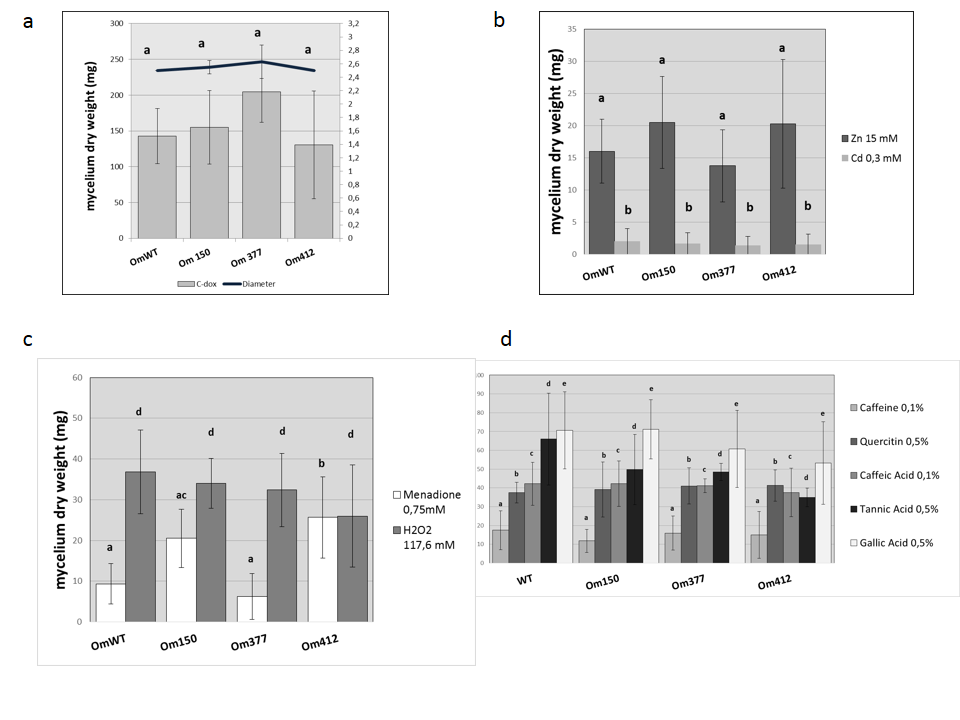


**(A)**

**(D)**

**(C)**

**(B)**

**Supplementary Figure 5. OmΔSSP1 mutants do not show impaired growth under stressful conditions.** The *O. maius* WT and the OmΔSSP mutants growth rate were investigated in (**A)** control medium, (**B**) in the presence of 0.3 mM Cd and 15 mM Zn, (**C**) in the presence of H_2_O_2_ 117.6 mM and menadione 0.75 mM and (**D)** in the presence of caffeine 0.1%, tannic acid 0.5%, gallic acid 0.5%, quercetin 0.5% and caffeic acid 0.1% . Fungal dry biomasses were measured after 30 days of growth. Bars represent the mean ±SD, n=5 (each biological replicate). Different letters indicate statistically significant difference (p < 0.05) (ANOVA, Tukey’s post hoc test).

## Supplementary Tables

**Table S1** Results of the RNA-Seq experiment: summary of reads alignments to the *O. maius* genome

| **Samples** | **trimmed reads** | **not aligned** | **aligned** | **% aligned** |
| --- | --- | --- | --- | --- |
| **Oidma-FLM1** | 52297206 | 11668722 | 40628484 | 77,7 |
| **Oidma-FLM2** | 59135170 | 13425192 | 45709978 | 77,3 |
| **Oidma-FLM3** | 40110112 | 8728011 | 31382101 | 78,2 |
| **Oidma-MYC1** | 57687306 | 56343363 | 1343943 | 2,3 |
| **Oidma-MYC2** | 42932494 | 41905372 | 1027122 | 2,4 |
| **Oidma-MYC3** | 72438460 | 71062609 | 1375851 | 1,9 |

**Table S2** List of the primers used in this study

| **Primer Name** | **5’-3’ sequence** | **Primer Use** |
| --- | --- | --- |
| ***Validation of*** ***gene expression*** | |  |
| *OmEF1α_F* | CGTCGTTATCGGCCACGTC | RT-qPCR |
| *OmEF1α_R* | TGACATAGTACCTGGGGGTCTCG |  |
| *OmβTub_F* | GTCTCCATGAAGGAGGTTGAGG |  |
| *OmβTub_R* | CAGAGAGCGGTCTGGACGTTGT |  |
| *OmSSP1_F* | CGCGGCTACTCCTCTCAACG |  |
| *OmSSP1_R* | TGGTTGTTGTCGCAGGTGGA |  |
| *OmSSP2_F* | GCACAGGCTCTTGCTGTTGC |  |
| *OmSSP2_R* | GACGAACGACCGCGTACTGA |  |
| *OmSSP3_F* | TCGTCGATTCCGTCACCAAA |  |
| *OmSSP3_R* | GTCCGTTCCGGCATTAGTGG |  |
| *OmSSP4_F* | GCCCTGCTAGCGCTTGCTTC |  |
| *OmSSP4_R* | CCCAATGCTGGGGTTGGATT |  |
| *OmSSP5_F* | CGCTTCTCTCATCGCCCTTC |  |
| *OmSSP5_R* | TTCTCTTCTACATGGCCCAGATCA |  |
| *OmSSP6_F* | GGCCGACTGGAGCGTTTATG |  |
| *OmSSP6_R* | CGGTGGAAGACTGGCCTTGT |  |
| *OmSSP7_F* | CCCCTCAGCAGCTCACATCA |  |
| *OmSSP7_R* | GGATGGGCAAAGGCTGTCAC |  |
| ***Construction of disruption pCAMBIA vector*** | |  |
| PreOmSSP1f_XmaIT | CCCGGGAGCATCTCTTACGGCCAAGA | OmSSP1 5’ flanking sequence production |
| PreOmSSP1rHindIIIT | AAGCTTCGATCAAATCCATCTAGCC |  |
| PostOmSSP1f_BglIIT | AGATCTAAGGACGCCACAAGACAAAT | OmSSP1 3’ flanking sequence production |
| PostOmSSP1r_HpaIT | GTTAACATACCGGATGCTGAGGTGAG |  |
| ***PCR screening of O. maius OmSSP1 null-mutants*** | |  |
| OmSSP1r | CAGACCTGCAAGCGTGACTA | OmΔSSP1 mutant screening |
| OmSSP1f | TGTTCCAGTGCGCGAGATGTA |  |
| Hyg2r | GCAGTTCGGTTTCAGGCAGG |  |
| Hyg4f | ATCTGTAGGGCGTCCAAATATC |  |
| ***PCR validation of vector insertion site*** | | PCR on flanking region on OmΔSSP1 candidates |
| PreOmSSP1f3 | GCTGTACCGGTGACCTAGGG |  |
| Hyg 3f | GACTGAGGAATCCGCTCTTG |  |
| PostOmSSP1r3 | GTTTAGCCTTGTCGCAAAA |  |
| Hyg6r | GCCAAAGGACCCTCTATGCTC |  |
| PreOmSSP1r2 | GATGCGATCGATCACTGAGA |  |

**Table S3** Sequences used to build the phylogenetic tree, for alignments and biochemical analysis (see the Excel file).

**Table S4 (a)** List and bioinformatics features of the 445 SSPs annotated in the *O. maius* genome; **(b)** list and bioinformatics features of the 90 symbiosis induced SSPs in *O. maius.* For each of them the number of orthologous genes found in the genome of other 59 fungi taxonomically and ecologically distinct is also reported; **(c)** selected SSPs orthologous genes found in the genome of other 59 fungi taxonomically and ecologically distinct; **(d)** names and codes of the 60 fungal species used for the genomic comparative analyses (see the Excel file).

**Table S5** Biochemical features of OmSSP1, of the four *O. maius* hydrophobins and of class I and II type hydrophobins

| **Name** | **Organism** | **Amino acid N** | **Molecular weight** | **GRAVY** | **β-sheet propensity** | **α-helical propensity** | **Intrinsic aggregation propensity** |
| --- | --- | --- | --- | --- | --- | --- | --- |
| OmSSPb1 | *O. maius* | 93 | 9241.59 | 0.531 | 333.8 | 339.4 | -1.3 |
| OmHydr.1 |  | 120 | 11603.42 | 0.622 | 396.7 | 419.4 | -4.2 |
| OmHydr.2 |  | 91 | 9484.08 | 0.763 | 313.1 | 335.7 | -5.5 |
| OmHydr.3 |  | 94 | 9587.26 | 0.619 | 418.9 | 445.9 | -4.8 |
| OmHydr.4 |  | 126 | 12577.32 | 0.316 | 333.7 | 344.7 | -4.2 |
| EAS (Type I Hydrophobin) | *N. crassa* | 91 | 9063.46 | 0.485 | 311.4 | 329.1 | -4.77 |
| HFBI (Type II Hydrophobin) | *T. reesei* | 97 | 9874.43 | 0.420 | 336.5 | 356.1 | -4.8 |
|  |  |  |  |  |  |  |  |
| Maximum values |  |  |  | 0,957 | 890 | 917 | -8 |
| Minimum values |  |  |  | -0,049 | 313 | 335 | -1 |
